# Supplementary material for: Light-Photoreceptors and Proteins Related to Monilinia laxa Photoresponses
Source: J Fungi (Basel). 2021 Jan 7;7(1):32. doi: 10.3390/jof7010032 (PMC7827745; doi:10.3390/jof7010032)
Supplement: Supplementary file 1 [file jof-07-00032-s001.zip › jof-1031713 suppl/Figure S1.pdf]

CLUSTAL W (1.81) multiple sequence alignment

```
B.cinerea_Bcin07g05880    ATGTACCATTCGAATCACGACGCGCTCCCAATTCATCAGCCTTCTGATGGCAGAACCATT
5L_VEL4                   ATGTACCATTCGAATCGCGACGCGCTTCCAATTTACCAGCCATCTGATGATAGGCCCACA
8L_VEL4                   ATGTACCATTCGAATCGCGACGCGCTTCCAATTTACCAGCCATCTGATGATAGGCCCACA
25L_VEL4                  ATGTACCATTCGAATCGCGACGCGCTTCCAATTTACCAGCCATCTGATGATAGGCCCACA
Mlax316_EYC80_007367      ATGTACCATTCGAATCGCGACGCGCTTCCAATTTACCAGCCATCTGATGATAGGCCCACA
                          **************** ********* ****** * ***** *******  **  ***

B.cinerea_Bcin07g05880    AGCTATGGACGAGAGCCGTTACCGTACCACGACATCGGCGATGACAGGGCGCAAATGCCG
5L_VEL4                   AGCTTTGGGAGGGAGCCACTCCCATATCGCGACAATGGCGATGACAGGGCGCAGATGCCA
8L_VEL4                   AGCTTTGGGAGGGAGCCACTCCCATATCGCGACAATGGCGATGACAGGGCGCAGATGCCA
25L_VEL4                  AGCTTTGGGAGGGAGCCACTCCCATATCGCGACAATGGCGATGACAGGGCGCAGATGCCA
Mlax316_EYC80_007367      AGCTTTGGGAGGGAGCCACTCCCATATCGCGACAATGGCGATGACAGGGCGCAGATGCCA
                          **** ***  * *****  * ** ** * *****  ***************** *****

B.cinerea_Bcin07g05880    AATTTCAATCCCCCCAGACTTCCTCCCATATCTTCATTGCTATCCTCTTTACCACCGGAA
5L_VEL4                   AATTTCAATTCCTTAAGACTCCCACCTATATCTTCATTGCTATCATCTCTACCGCCTGAA
8L_VEL4                   AATTTCAATTCCTTAAGACTCCCACCTATATCTTCATTGCTATCATCTCTACCGCCTGAA
25L_VEL4                  AATTTCAATTCCTTAAGACTCCCACCTATATCTTCATTGCTATCATCTCTACCGCCTGAA
Mlax316_EYC80_007367      AATTTCAATTCCTTAAGACTCCCACCTATATCTTCATTGCTATCATCTCTACCGCCTGAA
                          ********* **   ***** ** ** ***************** *** **** ** ***

B.cinerea_Bcin07g05880    CAAGTTGAGGAGGGTCAGACGTTTGATGAGTCGTATCGTCCGCCATCACAAGGACCACGG
5L_VEL4                   CAAGTGGAAGAAAGTCAAGGGTTCGAAGAATCATATCGCCCGCCATCACAAGGACCACGG
8L_VEL4                   CAAGTGGAAGAAAGTCAAGGGTTCGAAGAATCATATCGCCCGCCATCACAAGGACCACGG
25L_VEL4                  CAAGTGGAAGAAAGTCAAGGGTTCGAAGAATCATATCGCCCGCCATCACAAGGACCACGG
Mlax316_EYC80_007367      CAAGTGGAAGAAAGTCAAGGGTTCGAAGAATCATATCGCCCGCCATCACAAGGACCACGG
                          ***** ** **  ****   *** ** ** ** ***** *********************

B.cinerea_Bcin07g05880    TCTCCAGTAGCATTGAACAGGCCAATGTACCTACATCAGCAACAACGTAGTCCTCGGGAG
5L_VEL4                   TCTCCATTGGCATTGGACAGGCCAATGTACCTGCATCAGCAACAACGTAGTCCGCGGGAG
8L_VEL4                   TCTCCATTGGCATTGGACAGGCCAATGTACCTGCATCAGCAACAACGTAGTCCGCGGGAG
25L_VEL4                  TCTCCATTGGCATTGGACAGGCCAATGTACCTGCATCAGCAACAACGTAGTCCGCGGGAG
Mlax316_EYC80_007367      TCTCCATTGGCATTGGACAGGCCAATGTACCTGCATCAGCAACAACGTAGTCCGCGGGAG
                          ****** * ****** **************** ******************** ******

B.cinerea_Bcin07g05880    CTCGAACGCACTTTTAAGCACTCCCAGAGCCCGTTATCTCCATACTCGACATGTCCACCG
5L_VEL4                   CTTGAACTTTCCTTTAAGCATTCCCAGAGCCCAATTACTACATACTCGAGCGGTCCGCCA
```

```
8L_VEL4                   CTTGAACTTTCCTTTAAGCATTCCCAGAGCCCAATTACTACATACTCGAGCGGTCCGCCA
25L_VEL4                  CTTGAACTTTCCTTTAAGCATTCCCAGAGCCCAATTACTACATACTCGAGCGGTCCGCCA
Mlax316_EYC80_007367      CTTGAACTTTCCTTTAAGCATTCCCAGAGCCCAATTACTACATACTCGAGCGGTCCGCCA
                          ** ****   * ******** ***********  *  ** *********   **** ** 

B.cinerea_Bcin07g05880    ACACCCATGTCAGATGTATTTGCGACTAGTGAAGGTTCTGTCGCAGCTCATCACCACGGT
5L_VEL4                   ACACCAATGTCCGATGTGTTCGCTACCAGGGAAGGATCCGTCGCAACTCATTACCACGGT
8L_VEL4                   ACACCAATGTCCGATGTGTTCGCTACCAGGGAAGGATCCGTCGCAACTCATTACCACGGT
25L_VEL4                  ACACCAATGTCCGATGTGTTCGCTACCAGGGAAGGATCCGTCGCAACTCATTACCACGGT
Mlax316_EYC80_007367      ACACCAATGTCCGATGTGTTCGCTACCAGGGAAGGATCCGTCGCAACTCATTACCACGGT
                          ***** ***** ***** ** ** ** ** ***** ** ****** ***** ********

B.cinerea_Bcin07g05880    CCGTTTGCCCCTCCCAGGTATGTTATTTTGGTATCCATTGTCAATGGATGAAGGCTCATT
5L_VEL4                   CCATTTGCCCCTCCCAGGTATGTTATTTTGGTATCCATTGTCAATGGATGAAGGCTCATT
8L_VEL4                   CCATTTGCCCCTCCCAGGTATGTTATTTTGGTATCCATTGTCAATGGATGAAGGCTCATT
25L_VEL4                  CCATTTGCCCCTCCCAGGTATGTTATTTTGGTATCCATTGTCAATGGATGAAGGCTCATT
Mlax316_EYC80_007367      CCATTTGCCCCTCCC---------------------------------------------
                          ** ************

B.cinerea_Bcin07g05880    GTACTCAAAGGGCTCCGCCACCACTCCCCCCTCAGCCAGAATGTAGACTGGTCGTCATGC
5L_VEL4                   ATGCTCCAAGGGCTCCGCAACCA---CAACCACAGCCAGAATGCAAGCTGGTTGTAATGC
8L_VEL4                   ATGCTCCAAGGGCTCCGCAACCA---CAACCACAGCCAGAATGCAAGCTGGTTGTAATGC
25L_VEL4                  ATGCTCCAAGGGCTCCGCAACCA---CAACCACAGCCAGAATGCAAGCTGGTTGTAATGC
Mlax316_EYC80_007367      --------AGGGCTCCGCAACCA---CAACCACAGCCAGAATGCAAGCTGGTTGTAATGC
                                  ********** ****   *  ** *********** *  ***** ** ****

B.cinerea_Bcin07g05880    AACAGCCAATATCCGCACGAGCTTGTGGATACGGAGACAAGGACCGTCGAATGATAGACC
5L_VEL4                   AACAACCTATCTCCGCCAGAGCTTGTGGGTATGGAGACAAGGATAGGCGAATGATAGATC
8L_VEL4                   AACAACCTATCTCCGCCAGAGCTTGTGGGTATGGAGACAAGGATAGGCGAATGATAGATC
25L_VEL4                  AACAACCTATCTCCGCCAGAGCTTGTGGGTATGGAGACAAGGATAGGCGAATGATAGATC
Mlax316_EYC80_007367      AACAACCTATCTCCGCCAGAGCTTGTGGGTATGGAGACAAGGATAGGCGAATGATAGATC
                          **** ** ** *****  ********** ** ***********  * *********** *

B.cinerea_Bcin07g05880    CGCCCCCAATTCTGACGCTCGAAGTATTTGATAAGGATACAGGTCAGGCTGTCAATCCAA
5L_VEL4                   CACCGCCAATTTTGACTCTCGAAGTATTTGATAAAGATACCGGCCAGGAGGTCAACTCAA
8L_VEL4                   CACCGCCAATTTTGACTCTCGAAGTATTTGATAAAGATACCGGCCAGGAGGTCAACTCAA
25L_VEL4                  CACCGCCAATTTTGACTCTCGAAGTATTTGATAAAGATACCGGCCAGGAGGTCAACTCAA
Mlax316_EYC80_007367      CACCGCCAATTTTGACTCTCGAAGTATTTGATAAAGATACCGGCCAGGAGGTCAACTCAA
                          * ** ****** **** ***************** ***** ** ****  *****  ***
```

```
B.cinerea_Bcin07g05880    AGGAGAGAAAGAATGTCTTGAGTAAATACATGCCGATTGTGCATTGCACACTATGGAATC
5L_VEL4                   AGGAGAGAAAGAAGGTTTTGAACAGGTTCATGCCTATAGTGCATTGCACGCTGTGGAATC
8L_VEL4                   AGGAGAGAAAGAAGGTTTTGAACAGGTTCATGCCTATAGTGCATTGCACGCTGTGGAATC
25L_VEL4                  AGGAGAGAAAGAAGGTTTTGAACAGGTTCATGCCTATAGTGCATTGCACGCTGTGGAATC
Mlax316_EYC80_007367      AGGAGAGAAAGAAGGTTTTGAACAGGTTCATGCCTATAGTGCATTGCACGCTGTGGAATC
                          ************* ** ****  *  * ****** ** *********** ** *******

B.cinerea_Bcin07g05880    CCTACACAAATTGCGAAGATGACAAGATCGAGGGAAGTAGTGACCGTAGAAATCAAAGGA
5L_VEL4                   CATACACGAATTGCGAGGACGACAAAATTGAGGGAAGTAGTGACCGTCGAAATCAGAGGA
8L_VEL4                   CATACACGAATTGCGAGGACGACAAAATTGAGGGAAGTAGTGACCGTCGAAATCAGAGGA
25L_VEL4                  CATACACGAATTGCGAGGACGACAAAATTGAGGGAAGTAGTGACCGTCGAAATCAGAGGA
Mlax316_EYC80_007367      CATACACGAATTGCGAGGACGACAAAATTGAGGGAAGTAGTGACCGTCGAAATCAGAGGA
                          * ***** ******** ** ***** ** ****************** ******* ****

B.cinerea_Bcin07g05880    GGATGGTAGGAACGATGGTGTGTAACGGTTTCAAAGCACTAGATATTCATGGCGATGAGC
5L_VEL4                   GAATGGTAGGAACCATGGTCTGTAACGGCTTCAAGGCATTAGATCCTTCTGGCGATGAGA
8L_VEL4                   GAATGGTAGGAACCATGGTCTGTAACGGCTTCAAGGCATTAGATCCTTCTGGCGATGAGA
25L_VEL4                  GAATGGTAGGAACCATGGTCTGTAACGGCTTCAAGGCATTAGATCCTTCTGGCGATGAGA
Mlax316_EYC80_007367      GAATGGTAGGAACCATGGTCTGTAACGGCTTCAAGGCATTAGATCCTTCTGGCGATGAGA
                          * *********** ***** ******** ***** *** *****  *  **********

B.cinerea_Bcin07g05880    GATTCTTCTTCACTTTCGCCGATTTGTCTGTTCGATTCCCGGGTGACTATCAATTGAAAT
5L_VEL4                   GGTTCTTTTTCACTTTTGCCGACTTGTCCGTTCGGTTCCCAGGTGATTATCAATTGAAAT
8L_VEL4                   GGTTCTTTTTCACTTTTGCCGACTTGTCCGTTCGGTTCCCAGGTGATTATCAATTGAAAT
25L_VEL4                  GGTTCTTTTTCACTTTTGCCGACTTGTCCGTTCGGTTCCCAGGTGATTATCAATTGAAAT
Mlax316_EYC80_007367      GGTTCTTTTTCACTTTTGCCGACTTGTCCGTTCGGTTCCCAGGTGATTATCAATTGAAAT
                          * ***** ******** ***** ***** ***** ***** ***** *************

B.cinerea_Bcin07g05880    TCAGATTGACGTTAATCGACCCAGCTGCAATGGGGAAAGGTCAAAGAAACGGGATTATGA
5L_VEL4                   TCAGGTTAACGTTGATTGATCCAGCGGGAATGGGAAAGGGTCAGAGGAACGGAATCATGA
8L_VEL4                   TCAGGTTAACGTTGATTGATCCAGCGGGAATGGGAAAGGGTCAGAGGAACGGAATCATGA
25L_VEL4                  TCAGGTTAACGTTGATTGATCCAGCGGGAATGGGAAAGGGTCAGAGGAACGGAATCATGA
Mlax316_EYC80_007367      TCAGGTTAACGTTGATTGATCCAGCGGGAATGGGAAAGGGTCAGAGGAACGGAATCATGA
                          **** ** ***** ** ** ***** * ****** ** ***** ** ***** ** ****

B.cinerea_Bcin07g05880    GTCCATTGTTAAGCAATATCTTTAAGGTACATAATGCCAAAGACTTCGAGGGGATGAGGC
5L_VEL4                   GCCCATTACTCAGCAATATCTTCAAGGTCCATAATGCGAAAGACTTTGAGGGCATGAGAC
8L_VEL4                   GCCCATTACTCAGCAATATCTTCAAGGTCCATAATGCGAAAGACTTTGAGGGCATGAGAC
```

```
25L_VEL4                  GCCCATTACTCAGCAATATCTTCAAGGTCCATAATGCGAAAGACTTTGAGGGCATGAGAC
Mlax316_EYC80_007367      GCCCATTACTCAGCAATATCTTCAAGGTCCATAATGCGAAAGACTTTGAGGGCATGAGAC
                          * *****  * *********** ***** ******** ******** ***** ***** *

B.cinerea_Bcin07g05880    CTAGTTCGGATCTGGCGAAATCTCTTGTTAGCCAGGGCTATCCGAATATTCTCAAGATTA
5L_VEL4                   CCAGCTCAGACTTGGCCAAATCTCTTGTTAGCCAAGGCTATCCCAATATTCTCAAGATCA
8L_VEL4                   CCAGCTCAGACTTGGCCAAATCTCTTGTTAGCCAAGGCTATCCCAATATTCTCAAGATCA
25L_VEL4                  CCAGCTCAGACTTGGCCAAATCTCTTGTTAGCCAAGGCTATCCCAATATTCTCAAGATCA
Mlax316_EYC80_007367      CCAGCTCAGACTTGGCCAAATCTCTTGTTAGCCAAGGCTATCCCAATATTCTCAAGATCA
                          * ** ** **  **** ***************** ******** ************** *

B.cinerea_Bcin07g05880    AGAAAGGAAATGCGAAAACAATTGCAATGGTTGGCCAAGGG------------------G
5L_VEL4                   AAAAGGGCAATGCGAAGACCATTGCAATGGTTGGCCAAGGAGCTCAT------GATGACG
8L_VEL4                   AAAAGGGCAATGCGAAGACCATTGCAATGGTTGGCCAAGGAGCTCATGATGACGATGACG
25L_VEL4                  AAAAGGGCAATGCGAAGACCATTGCAATGGTTGGCCAAGGAGCTCAT------GATGACG
Mlax316_EYC80_007367      AAAAGGGCAATGCGAAGACCATTGCAATGGTTGGCCAAGGAGCTCAT------GATGACG
                          * ** ** ******** ** ********************                   *

B.cinerea_Bcin07g05880    ACGACGAGGATGACGATGATGAAATGGGAGGAA------TGTAA----------------
5L_VEL4                   ATGACGATGATGATGATGATGAGATGGGAGGTA------TGTAAAGCAGTT---------
8L_VEL4                   ATGACGATGATGATGATGATGAGATGGGAGGTA------TGTAAAGCAGTT---------
25L_VEL4                  ATGACGATGATGATGATGATGAGATGGGAGGTA------TGTAAAGCAGTT---------
Mlax316_EYC80_007367      ATGACGATGATGATGATGATGAGATGGGAGAGAATCTAGTGAAAAGTGTCTTGGAGAGTG
                          * ***** ***** ******** *******  *      ** **

B.cinerea_Bcin07g05880    ----------------------------------------------------
5L_VEL4                   ----------CTTGCTGGGTATACTGATACCATGATAGCCAAGAGAATCTAG
8L_VEL4                   ----------CTTGCTGGGTATACTGATACCATGATAGCCAAGAGAATCTAG
25L_VEL4                  ----------CTTGCTGGGTATACTGATACCATGATAGCCAAGAGAATCTAG
Mlax316_EYC80_007367      AGGACGAAGACTTACGAGCTTTGCTGTTTGCATTGTATTGA-----------
```

Figure S1. Alignment of *vel4* gene from *M. laxa* isolates 5L, 8L, and 25L with *velvet4* from *B. cinerea* B05.10 and *M. laxa* Mlax316 using Clustal W 1.81. Asterisks are placed underneath conserved nucleotide.
