# Supplementary material for: Light-Photoreceptors and Proteins Related to Monilinia laxa Photoresponses
Source: J Fungi (Basel). 2021 Jan 7;7(1):32. doi: 10.3390/jof7010032 (PMC7827745; doi:10.3390/jof7010032)
Supplement: Supplementary file 1 [file jof-07-00032-s001.zip › jof-1031713 suppl/Table S1.pdf]

Table S1. Quantitative real-time polymerase chain reaction (RT-qPCR) primer set for the analysis of genes implicated in photoresponses and potential photoreceptors of *M. laxa* and primer set for *velvet4* sequencing.

**Gene**		**Sequence (5'-3')**	**Product size (bp)**
MlCRY1	F	GGCTTGGGTTGATGAACTACG	236
	R	TCCATCTATCGCCACCATGTG	
MlCRY2	F	TTCTGGCGAAACATCTGCTG	232
	R	CCCTTCACATCCCTCAACTCC	
MlWC1	F	ACGTAGTGGTAGTAAGGATGGG	225
	R	TTACCTCCCGCACCATCATTC	
MlWC2	F	AACTGGGCACATAAGCAACC	225
	R	ATCCACACCCTCATATCGCATC	
MlOPS1	F	TCTTCGCTGCTTACCACAAC	239
	R	AGAATCAACCGACCATGTGC	
MlOPS2	F	CTCGATGTGCTAGCCAAGATTG	217
	R	CGTTGGCAGGATTATTCGAGTG	
MlPHY1	F	AGCACCATCGAATGAAACGC	239
	R	CTATCACGGGTCTTTCATCAGC	
MlPHY2	F	AGAGGGATCATCCTGAATTGCG	237
	R	AAGAACCACCCACCATGTTC	
MlPHY3	F	CTATTGGAAGAGCGCCTGAG	225
	R	TAGACGACCATAGACGCTAGC	
MlVEL1	F	ACAGCCATGTCTCCACTTGC	216
	R	ATTGTGGAGCGGTTCTTGAG	
MlVEL2	F	ACTCATCGTGCTCCGTCAAC	218
	R	ATCGTCATCACCTCGGCTAC	
MlVEL3	F	TATCACCCGCAATCGCAAGG	234
	R	TCCAGGCAGTGTGTCAAAGG	
MlVEL4	F	GGTCTGTAACGGCTTCAAGG	222
	R	CTCATGCCCTCAAAGTCTTTCG	
Ml_HistoneH3	F	TCCGTCGTTACCAAAAGTCG	230
	R	GGCGAGTTGGATGTCCTTAG	
MlRPL5	F	ACACGTTGCTGAGTACATGG	240
	R	TCTTCCTTGGTCAACTTGCG	
Sequencing of *velvet4*	F	TTCAGCAACTACTGGCACC	1516
	R	AACTCCTGCCTTCAAGGACC	
